# 

**Published:** 1871-11

**Authors:** 


### Contents of November Number.

### ORIGINAL DEPARTMENT.

ART. 1. Clinical Remarks on Erysipelas. By Prof. Thos. F. Rochester, in the Hospital of the Sisters of Charity. Reported by Edward N. Brush, member of the class ... 121
ART. 2. Hospital Notes. By C. C. F. Gay, M. D., Surgeon to the Buffalo General Hospital ... 124
ART. 3. Clinical Lectures upon Surgical Cases in the Buffalo Hospital of the Sisters of Charity. By Prof. J. F. Miner. Reported by Louis Brecht, member of the class of 1871-'72 ... 127

### MISCELLANEOUS.

An Oakum Respirator. By Somerville Oliver, M.D. ... 130
Short Notes of Post-Mortem Examinations.—Vienna ... 131
The Treatment of Drunkards ... 133
On Sesquichloride of Iron as a Prophylactic of Acute Rheumatism. By Dr. Anstie ... 134
On the Physiological Action of Bromide of Potassium ... 135
A New and Important Therapeutic Property of Quinine ... 139
The True and False Cundurango ... 140
Resolutions of the New York Academy of Medicine ... 142
Resolutions of the Albany County Medical Society relative to Abortion ... 143
Where shall we send our Consumptive Patients? ... 144
The Value of Meat Extract ... 145
The Action of Mercury in Children. By W. L. Nichol, M.D ... 145
Case of remarkable Presentation of the Fœtus in Utero ... 148
Relapsing Fever in Carlisle ... 149
Treatment of Pneumonia ... 150
Pathology of the Prostate Gland ... 150
Undulatory Hypothesis Applied to Sensation ... 151
Fatal Salivation from Bichloride of Mercury ... 151

### EDITORIAL DEPARTMENT.

Bromide Substituted for Iodide of Potassium ... 152
Alumni Association of Buffalo ... 153
Rush Medical College ... 153

BOOKS REVIEWED:

Electrolysis and its Application to the Treatment of Disease. By A. D. Rockwell, A.M., M.D ... 154
Contributions to Practical Laryngoscopy. By A. Ruppaner, A.M., M.D 155
Introductory Lecture to the Course on Pathological Anatomy at the University of Philadelphia. By Joseph G. Richardson, M.D ... 155
The First Annual Report of the Board of the Free Medical and Surgical Dispensing Association of Buffalo ... 155
Practical Therapeutics. By Edward John Warring, M.D ... 155
Prolapsus Uteri. By Thomas Addis Emmett, M.D ... 157
The Management of Infancy, Physiological and Moral. By Andrew Combe, M.D. ... 157
Retrospect of Uterine Pathology and Therapeutics in the United States, By Henry Miller, M D. ... 157
Anatomical Remembrancer, or Complete Pocket Anatomist. By C E. Isaacs, M.D ... 157
Handy-Book of the Treatment of Women's and Children's Diseases. By Emil Dillnberger ... 158
Essentials of the Principles and Practice of Medicine. By Henry Hartshorne, M.D. ... 158

Notice ... 158
Detroit Medical College ... 158
New York Observer ... 159
Books and Pamphlets Received ... 159

---

**THE BUFFALO**

